# Reablement through time and space: a scoping review of how the concept of ‘reablement’ for older people has been defined and operationalised

**DOI:** 10.1186/s12877-020-01958-1

**Published:** 2021-01-15

**Authors:** Amy Clotworthy, Sasmita Kusumastuti, Rudi G. J. Westendorp

**Affiliations:** Department of Public Health, University of Copenhagen, Bartholinsgade 6Q, 1014 Copenhagen K, Denmark

**Keywords:** Reablement, Rehabilitation, Restorative care, Ageing, Homecare, Health services, Activities of daily living, Literature review

## Abstract

**Background:**

While the field of rehabilitation has determined a common definition of professional practice, legislators and healthcare professionals in various Western countries have struggled to reach consensus about how the newer offer of ‘reablement’ should be organised, operationalised, and understood as a health service for older adults. International research indicates that there is confusion, ambiguity, and disagreement about the terminology and the structure of these programmes, and they may not be adequately supporting older people’s self-identified goals. Could an analysis of the concept’s genealogy illuminate how reablement can be more effective and beneficial in theory *and* in practice?

**Methods:**

We conducted a qualitative and quantitative scoping review to determine how reablement has developed through time and space. Eligible articles (*N*=86) had to focus on any of the defined features of current reablement programmes; there were no restrictions on study designs or publication dates. In articles published from 1947 to 2019, we identified themes and patterns, commonalities, and differences in how various countries described and defined reablement. We also performed an analysis using computer software to construct and visualise term maps based on significant words extracted from the article abstracts.

**Results:**

The fundamental principles of reablement have a long history. However, these programmes have undergone a widespread expansion since the mid-2000s with an intention to reduce costs related to providing long-term care services and in-home assistance to growing older populations. Despite theoretical aspirations to offer person-centred and goal-directed reablement, few countries have been able to implement programmes that adequately promote older people’s goals, social involvement, or participation in their local community in a safe, culturally sensitive and adaptable way.

**Conclusions:**

Reablement is meant to support older people in attaining their self-defined goals to be both more physically independent at home and socially involved in their communities. However, until legislators, health professionals, and older people can collectively reach consensus about how person-centred reablement can be more effectively implemented and supported in professional home-care practice, it will be difficult to determine a conceptual description of reablement as a service that is unique, separate, and distinct from standard rehabilitation.

## Background

Population ageing raises significant questions about the socio-economic sustainability of increased human longevity, and this demographic shift has resulted in the promotion of ‘healthy ageing’ in many Western societies. While there is no universally accepted definition for ‘healthy ageing’ – or active, productive, or successful ageing – such concepts typically refer to individual or collective strategies used to optimise economic, social, and cultural participation throughout the life course [1]. The paradigm of ‘healthy ageing’ is often emphasised in legislation and social policies that target older people [2–5], particularly in high-income, industrialised Western societies that tend to valorise individualism and productivity. For example, during the International Federation on Ageing (IFA) Global Think Tank and Copenhagen Summit 2015/2016, attendees from many of these countries met to discuss ways to develop a solution-driven global public-health agenda that would further promote ‘healthy ageing’. One result of the summit was an agreed intention to advance “improved awareness of the value of a reablement approach and applied technology for an increasing ageing population” [6]. Here, reablement for the individual older person was defined as “an active process of (re) gaining skills and confidence in maintaining or improving function, or adapting to the consequences of declining function. It also supports the individual to remain socially engaged within the community context in a safe, culturally sensitive and adaptable way” [6].

Although this definition may seem straightforward, it is not universally accepted. This is because the concept of ‘reablement’ is relatively new and lacks regulation; thus, it is generally considered to be “poorly defined, with little understanding of what it looks like when achieved” [7]. Furthermore, while the related field of physical rehabilitation has long had a conceptual description to “foster the development of a common understanding of rehabilitation and its professions” [8], it appears that legislators and healthcare professionals both within and across various Western countries have been struggling to reach consensus about how reablement programmes should be defined, described, and structured. As such, there is ambiguity about what reablement is, what these programmes offer, and why (e.g., some may not specifically target older people; most are defined as being time-limited and home-based) as well as how reablement can best promote ‘healthy ageing’. Could an analysis of the concept’s genealogy illuminate how reablement can be more effective in both theory *and* in practice to benefit older people?

We performed a quantitative and qualitative exploration of how the concept of ‘reablement’ has developed over time in various countries to examine the common features and differences between these programmes at an international level. With our investigation of the term’s genealogy, this scoping review contributes insights and provides clarity to legislators, health professionals, and older people with regards to how these programmes can be organised, operationalised, and understood in practice – fundamentally, this clarification will enable them to collectively determine what ‘reablement’ looks like when it is achieved.

## Methods

On 25 March 2019, we searched Medline, Embase, CINAHL, and PsycInfo for all articles on reablement. There were no restrictions on publication dates. Due to the exploratory nature of our study and our main interest in reablement, the search term for all databases was “reablement OR re-ablement”. This resulted in papers published in English by researchers primarily working in high-income Western countries (as defined by the Organisation for Economic Co-operation and Development, International Monetary Fund, World Health Organization, and the United Nations). We examined the full texts of potentially eligible articles, and then used reference tracking to scrutinise reviews, updates, and protocols for additional potentially eligible articles.

### Article selection

Two authors (AC and SK) jointly examined all resulting articles for eligibility. Disagreements were resolved through consultation with a third reviewer (RGJW). For an article to be eligible, it had to focus on any of the most common aspects of ‘reablement’ as currently defined by most local government officials or health- or aged-care organisations:

- time-limited offer
- home-based intervention
- intervention implemented to improve a person’s functional ability and improve or develop their social participation (i.e., to support them in being more socially active).

The resulting original contribution had to be a complete research article, study protocol, or editorial published in an academic journal or book, and therefore not a conference abstract or poster. We did not apply restrictions on study designs (quantitative or qualitative) or publication dates.

### Qualitative analysis of the eligible articles

Starting with the earliest articles published, we examined the titles and abstracts for themes and patterns, commonalities, and differences in how ‘reablement’ was described and defined. In order to inform our analysis, we aimed to identify several shifts in terms of how reablement programmes have developed, which components they share, how reablement is organised and delivered as a service model, and how/why reablement is offered by healthcare authorities and to whom. This thematic analysis provided flexibility and allowed us to manage a large initial dataset; it was also an efficient way for us to determine the conceptual shifts in how reablement programmes have developed over time. Once we determined these predominant themes and patterns, we reviewed all of the relevant articles in full.

### Quantitative analysis of the eligible articles

To validate the findings from our qualitative analysis, we also performed a quantitative analysis of the eligible articles using VOS viewer software version 1.6.11 [9]. Here, we used the software’s text-mining functions to construct and visualise term maps based on scientific terms extracted from the eligible abstracts. A term map is a two-dimensional visualisation that represents networks of concurrent relations between scientific terms. The relatedness of terms is determined based on two or more terms occurring together in the abstracts. The smaller the distance between two terms, the stronger the terms are correlated with each other. Multiple terms with high co-occurrence are grouped together into clusters, and each cluster can therefore be seen as a confined intellectual topic. We used a full counting method, meaning that the number of occurrences of a term in a document was taken into account instead of only looking at the presence or absence of a term. Parts of the texts were tagged to identify verbs, nouns, adjectives, etc., and thereafter a filter selected all word sequences that consisted exclusively of nouns and adjectives. We also set a threshold of minimum five occurrences for a term to be included in our analysis. Out of all of the terms included, a relevance score was calculated and, based on this score, the most relevant terms were selected. The top 60% of the most relevant terms were visualised and coloured according to publication year. It is likely that some of the terms may appear across multiple time periods. However, each term is only visualised once, depending on the average publication year of articles in which the term was mentioned.

## Results

**Fig. 1:**
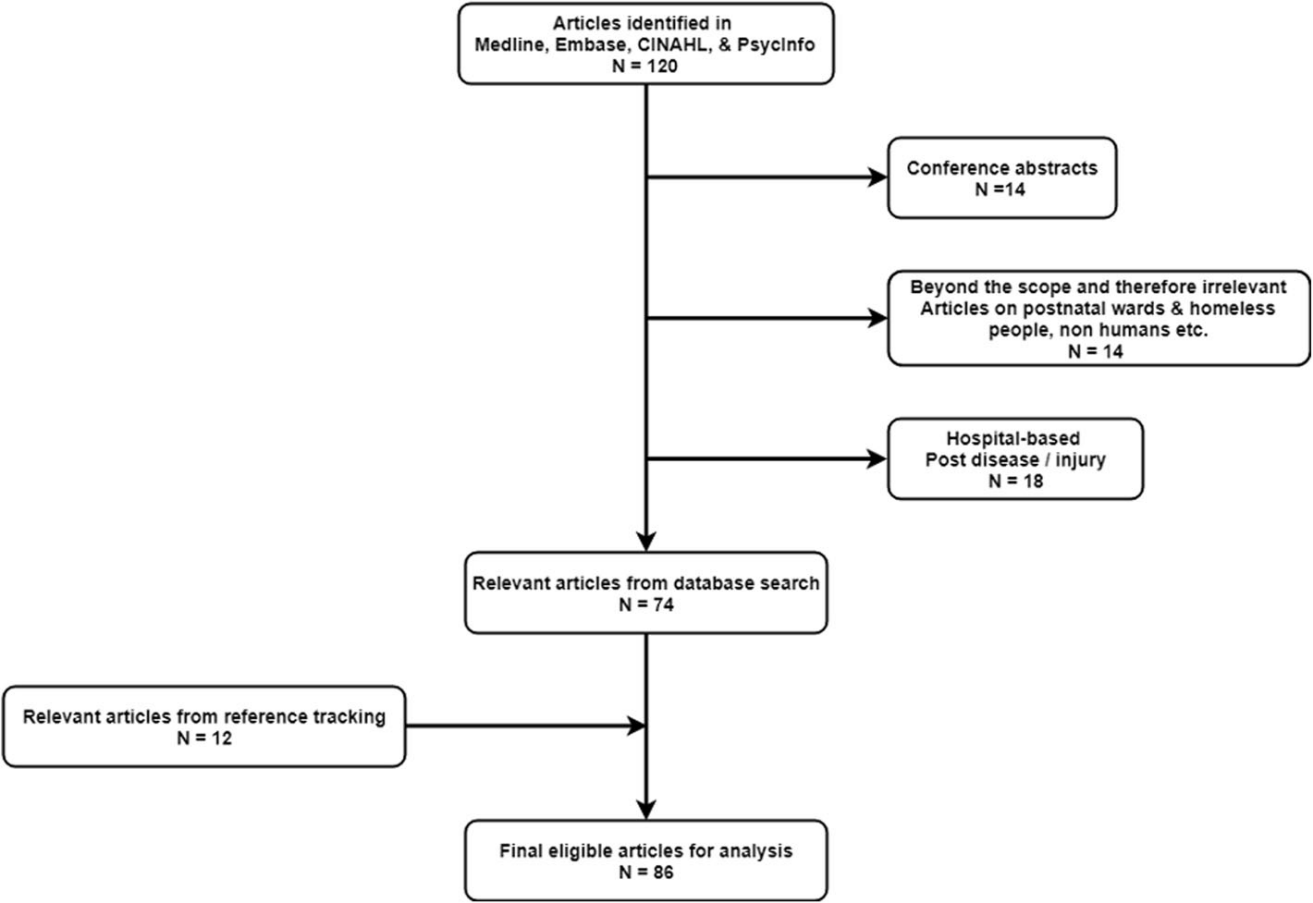
Flow Chart of Article Selection. This flow chart describes the selection process from articles identified (*N*=120) to final eligible articles for analysis (*N*=86)

Figure 1 presents a flow chart describing the selection of articles. Out of the 120 articles identified in our initial search in Medline, Embase, CINAHL, and PsycInfo databases, 14 were conference abstracts, 14 were beyond the scope and therefore irrelevant (e.g., articles on postnatal wards, homeless people, non-humans, etc.), and 18 were hospital-based post-disease/ -injury, which resulted in 74 relevant articles from the database search. We added another 12 relevant articles from reference tracking for a total of 86 eligible articles that were appropriate for the next stage of quantitative and qualitative analysis.

### Qualitative analysis

Based on the collected literature, we divided the conceptual shifts about reablement into the following categories: historical focus (pre-2000); the emergence of ‘restorative care’ for older people (2001–2005); the transition from ‘restorative care’ to ‘reablement’ (2006–2014); and the boom of reablement services (2015–2019).

#### Historical focus (pre-2000)

Our literature review indicated that the meaning and conceptualisation of ‘reablement’ has changed since the beginning of the twentieth century; in practice, it has both run parallel to and deviated from its sister practice, rehabilitation. Notably, after World War II, the emphasis of clinical rehabilitation began to broaden from a patient’s recovery of ambulation to a focus on “the comprehensive restoration of an individual’s physical, mental, emotional, vocational, and social capacities” [10, 11]. Around the same time, the word ‘reablement’ first entered the scholarly lexicon in an editorial published in *Physiotherapy* that was simply titled “Disablement and reablement” [12]. The author argued that the word ‘rehabilitation’ must be discarded in favour of ‘reablement’, which should focus on “making a disabled person able-bodied again” and restoring “a man to capacity for his former work, or, alternatively, what particular work he can be trained for” [12].

The publication of this editorial at the end of World War II suggests that the author’s conceptualisation of reablement was inspired by the need to support and train ‘dis-abled’ patients – most likely injured soldiers returning from the frontlines – in restoring their functional abilities and ‘re-settling’ them both at home and in the workplace. The term ‘reablement’ also started to be used to describe physiotherapeutic training for patients suffering from specific health conditions or diseases, such as cerebral palsy and rheumatoid arthritis [13, 14]. The first mention of reablement associated with “the heavily handicapped patient” was found in the title of a British journal article on the principles of rehabilitation in 1962 [15], and an article from 1975 discussed reablement in relation to functional activities and “resettlement in work and home” [16].

Throughout the 1960s and 1970s, there was a continued emphasis on the restoration of functional ability and “resettlement in work and home” [17–19] as well as ‘re-abling’ patients suffering from arthritis and other rheumatic diseases [20]. Few scholarly articles published in the 1980s and 1990s mentioned the term ‘reablement’, but a 1994 editorial in *Reviews in Clinical Gerontology* was the first to mention a focus on older people: “‘We are trying to improve this patient’s quality of life’ is the often stated goal of much therapeutic effort made for elderly people” [21], continuing that “the criteria for successful rehabilitation are often limited to independence in a limited range of basic activities of daily living. Reablement is most obviously concerned with reducing disability and the appropriate outcomes must be disability measures. ‘Resettlement’ is a more complex goal and implies the restoration of people to their own, or sometimes a new, environment” [21]. This article suggests that the term ‘reablement’ was still linked to ‘rehabilitation’ while ‘resettlement’ was understood as a different approach that included a focus on the environmental ‘restoration’ of older people.

#### The emergence of ‘restorative care’ for older people (2001–2005)

By the turn of the millennium, this idea of ‘resettlement’ and ‘restoration’ as a specific intervention for older people receiving homecare had transitioned into ‘restorative care’. In 2002, Tinetti et al. published what could be considered a landmark paper in the *Journal of the American Medical Association* (JAMA) titled: “Evaluation of restorative care vs usual care for older adults receiving an acute episode of home care”. The authors suggest that “a primary goal of health care for older, particularly multiply and chronically ill, persons should be to optimize function and comfort rather than solely to treat individual diseases” [22]. The restorative-care model that the authors described was based on principles adapted from geriatric medicine, nursing, rehabilitation, and goal attainment, which refers to the belief that people “are more likely to adhere to treatment plans if they are involved in setting goals and in determining the process for meeting these goals” [22].

The intervention included training relevant health professionals (i.e., homecare nurses, therapists, and home health aides) as well as reorganising this home-care staff “from individual care providers into an integrated, coordinated, interdisciplinary team with shared goals”, which required a “reorientation of the focus of the homecare team from primarily treating diseases and ‘taking care of’ patients toward working together to maximize function and comfort” [22]. Furthermore, it was built upon “the establishment of goals based on input from the patient, family, and home care staff, and agreement among this group on the process for reaching these goals” [22].

Although the authors cite several studies from the 1990s that focused on goal-setting in clinical medicine for the so-called ‘frail elderly’, this intervention appears to be the first well-defined programme that reflects the central aims of reablement as it is presently being operationalised and implemented worldwide; in other words, while the restorative-care model was not explicitly called ‘reablement’, it contained similar features and aims, and seems to have provided the philosophical foundation for many of today’s reablement programmes. Tinetti et al.’s research also built its findings on the authors’ earlier study, “The design and implementation of a restorative care model for home care” [23] as well as several previous articles that examined older people’s self-care and functionality with regards to performing activities of daily living (ADLs) in both hospital and home settings.

During this period, researchers in the United Kingdom also published a report that described 33 services that were part of the NHS Modernisation Agency’s Changing Workforce Programme project [24]. These services were “designed to prevent inappropriate hospital admissions, facilitate hospital discharge, and prevent premature or avoidable admissions to long-term care” [24] – and this included what the authors called ‘reablement services’. The summary of care roles outlines that this service focuses on providing “personal care, daily living skills, mobility and financial management (…); enablement rather than ‘doing’ for their services users; dedicated home workers providing personal care from an enabling perspective with rehabilitation skills; reablement to promote independence” [24].

#### The transition from ‘restorative care’ to ‘reablement’ (2006–2014)

The foundational aims of ‘restorative care’ services for older people seem to have transitioned to being called ‘reablement’ more broadly in the late-2000s, and our search identified 24 relevant articles from this time period. In the United Kingdom, this transition was reinforced by a retrospective longitudinal study of ‘homecare re-ablement’, “Research into the longer-term effects of reablement services” [25], which was undertaken for the Care Services Efficiency Delivery (CSED) Programme at the Department of Health. In the *Journal of Integrated Care*, it was stated that homecare reablement had been widely accepted in the UK, and referred to “services for people with poor physical or mental health to help them accommodate their illness by learning or re-learning the skills necessary for daily living” [26].

Other British researchers emphasised progress in developing “outcomes-focused services for older people and the factors that help and hinder this” [27]. The authors also described two small-scale, exploratory studies that examined the impact of home-care reablement on subsequent service use [28]. These articles distinguish ‘reablement’ as its own unique approach, describing it as a service that “aims to help people ‘do things for themselves’, rather than ‘having things done for them’. Home care reablement services therefore provide personal care, help with mobility and other practical tasks for a time-limited period” [28]. The authors further stated that user-identified outcomes are central to the reablement process, particularly with regards to personal care, daily living tasks, or social activities. They also pointed out that reablement services can be offered to adults of all ages in the UK, although they suggested that “even very elderly…users may regain skills and attitudes to help sustain them for a relatively long period” [28]. The same authors published a prospective longitudinal study and working paper that investigated the longer-term impacts of “home care re-ablement services” [29].

During this time period, a greater focus on professional practice was beginning to develop [30], and a professional column in *British Journal of Community Nursing* stated that reablement is “generally provided by local authorities as part of adult social care provision with a focus upon promoting self-care skills and rebuilding confidence” [31]. Reablement services targeted specifically at older people was also beginning to emerge in northern Europe – particularly, Finland. However, such programmes were termed “geriatric rehabilitation’ [32] and did not take place within the home setting. But, other than this difference, the Finnish intervention had aims similar to other reablement programmes; e.g., the goal was “to achieve and maintain functional independence, and to enable older people to remain community-dwelling” [32].

By 2011, British experts reiterated the burgeoning ‘reablement philosophy’, which states that “the focus is on restoring independent functioning rather than resolving health care issues, and on helping people to do things for themselves rather than the traditional home care approach of doing things for people that they cannot do for themselves” [33]. Other researchers claimed that “home-care re-ablement or ‘restorative’ services” should enable older people to live independently in the community, writing that “the assumption underlying re-ablement is that enhancing independence and practical skills reduces needs for ongoing service support” [34]. However, there remained a lack of consensus about the definition, organisation, and practices related to the overall concept of reablement. For example, a 2011 Taiwanese article discussed community hospital-based post-acute care (PAC) to improve functional ability amongst frail older patients [35], which appears to be the first time East Asian researchers mention the term.

A 2012 British article – which claimed to be the first in-depth study of the experiences of home-care reablement service users and carers – described reablement as “a short-term, intensive service that helps people to (re-) establish their capacity and confidence in performing basic personal care and domestic tasks at home, thereby reducing needs for longer term help” [36]. The authors noted that similar programmes were also being implemented in Australia and New Zealand. In Australia, the term ‘restorative care’ was being used to describe interventions with aims similar to many current reablement programmes. For example, the provision and nature of home-care services had developed a “new focus on activity, independence and successful ageing” and concluded that “a restorative approach to home care has significant advantages over the traditional approach aimed at maintenance and support only” [37]. However, the National Development Manager–Care and Communities for Age UK argued that reablement is “nowhere near as effective as it could be” because it lacks personalisation and “fails to appreciate what motivates people to make the substantial effort involved in regaining lost skills and abilities” [38].

By 2013, Australian researchers began to describe restorative home-care services as “short-term and aimed at maximizing a person’s ability to live independently. They are multidimensional and often include an exercise program to improve strength, mobility, and balance” and “the intervention should ultimately “create independence, improve self-image and self-esteem, and reduce the level of care required through the delivery of an individualized program” [39]. The linguistic transition from ‘restorative care’ to ‘reablement’ in Australia is clearly seen in a study that aimed “to determine whether older individuals who participated in a reablement (restorative) program rather than immediately receiving conventional home care services had a reduced need for ongoing support and lower home care costs” [40]. These authors also provided a straightforward definition of reablement in Australia; i.e., “an emphasis on capacity building (…) to maintain or promote a client’s capacity to live as independently as possible, with an aim of improving functional independence, quality of life, and social participation, (…and) an emphasis on a holistic, person-centered approach to care, which promotes clients’ wellness and active participation in decisions about care” [40].

However, it appears that a semantic debate was still occurring in Ireland and the UK. An Irish intervention protocol described ‘reablement’ (in scare-quotes) as “an innovative approach to improving home-care services for older adults in need of care and support or at risk of functional decline” [41], and the authors identified five essential defining criteria for an intervention to be called ‘reablement’: 1. participants must have an identified need for formal care and support, or are at risk of functional decline; 2. the intervention must be time-limited (typically 6–12 weeks) and intensive (e.g., multiple home visits); 3. the intervention must be delivered in the older person’s own home; 4. the intervention must focus on maximising independence; and 5. the intervention must be person-centred and goal-directed [41]. A 2013 British article described such programmes as “re-ablement or restorative homecare services that provide time-limited input aimed at reducing dependency in personal activities of daily living, and preventing or delaying the need for further homecare support” [42] while a 2014 Australian paper made the distinction between terminology in the different countries: “Restorative home-care services, or re-ablement home-care services as they are now known in the UK, aim to assist older individuals who are experiencing difficulties in everyday living to optimise their functioning and reduce their need for ongoing home care” [43].

Nearly all of the articles during this time period also cite a growing political interest in cost-savings. For example, although the emphasis in the UK remained on providing services to adults of all ages (not specifically older adults), the white paper, *Caring for Our Future: Reforming Care and Support,* provided a definition of reablement in which “users receive homecare but are supported to increase their ability to manage tasks independently, in order to reduce the amount of homecare they will require in the longer term” [44]. The authors further distinguished reablement from rehabilitation, stating that reablement services “adopt a social model of recovery rather than a medical model” [44]. During this time, there is also increased emphasis on developing professional practice; e.g., a critical literature review analysed evidence on the effectiveness and cost-effectiveness of occupational therapy interventions for older people in social-care services, including rehabilitation and reablement [45].

In Scandinavia, reablement programmes started to be offered in the 2000s; the first was established in Östersund Municipality, Sweden, in 1999 and – inspired by the Swedes’ positive experiences – soon began to develop in neighbouring Denmark (2008) and Norway (2012) under different names. The study protocol for a randomised controlled trial (RCT) of reablement in community-dwelling adults described it as “an approach to improve home-care services for older people needing care or experiencing functional decline. It is a goal-directed and intensive intervention, which takes place in the person’s home and local surroundings with a focus on enhancing performance of everyday activities defined as important by the person” [46]. A subsequent Danish article referred to the Nordic concept of ‘help to self-help’, which is based on “ways of providing help that involves the activation of older people, the aim being to enable them to manage as much as possible themselves” [47].

#### The boom of reablement services (2015–2019)

Our search identified 56 articles from this time period; the majority were published by researchers from Norway (*N*=19) and the United Kingdom (*N*=12), and they include both quantitative and qualitative studies conducted in these countries as well as a few collaborative studies conducted by researchers from different countries. There are no articles from Asia, South/Central America, or Russia and only one from continental Europe. If reablement-like programmes have been or are currently being offered in these regions, they are most likely called another name. We have summarised the regional studies and organised these results alphabetically by region in Table 1.

**Table 1:** Concept of Reablement in Various Geographic Regions between 2015 and 2019

Region (Country)	Concept of reablement between 2015 and 2019
Australasia (Australia/New Zealand)	• Australian research mentions that ‘reablement’, developed and formalised since the 1990s, has focused on goal-oriented cognitive rehabilitation to improve everyday functioning for people with dementia. Such interventions have been described as a form of reablement [7, 48–53]. • The book *Ageing in Australia* included a chapter “Care and Support for Older People” with a section on the more ‘typical’ reablement, defined as “(short-term) services for people with poor physical or mental health to help them accommodate their illness by learning or relearning the skills necessary for daily living” [54]. It was discussed as a person-centred service and emphasised the necessity to develop an aged-care workforce of professionals capable to do things *with* older persons and find optimal ways to engage family carers. • While restorative home care / reablement services for older adults have been “shown to be effective in reducing functional dependency and increasing functional mobility, confidence in everyday activities, and quality of life” [55], the literature often focuses on support workers and non-health professionals; e.g. to receive better training and improve collaboration [55], to promote health-behaviour change [56], and to develop the skills to work with complexity in community aged care [57]. • A trend towards developing better collaboration is evident in a paper about the feasibility of LifeFul, a relationship and reablement-focused “culture change program” in residential aged care [58]. However, the authors stated that one of the main challenges to successfully implementing reablement has been compliance by staff. • A critical review described reablement as “an emerging global practice model in community- and home-based care for older people” [59] and, although it is gaining acceptance worldwide, researchers and policy makers still have questions about what reablement means and how it is used in practice. They found nine essential features, the most predominant being the wish to improve the functionality of clients so they can continue to live in their own homes; the authors concluded that the under-representation of social connectivity for clients was regrettable [59]. • A New Zealand paper discussed benchmarking to assist the improvement of service quality in home support services for older people and stated that the country “has developed restorative home support services, very close to what in the United Kingdom are called reablement services, involving elements of goal facilitation, functional and repetitive ADL exercises, support worker training and enhanced supervision, health professional training, care management and comprehensive geriatric assessment” [60].
Continental Europe (Netherlands)	• A Dutch study protocol for a randomised controlled trial (RCT) on the effects, costs, and feasibility of the ‘Stay Active at Home’ reablement training programme for homecare professionals described reablement as home-care services that are “goal-oriented, holistic and person-centred taking into account the capabilities and opportunities of older adults” [61].
North America (Canada/United States)	• Despite the early implementation of reablement in the United States, our search did not result in any articles from this country during this time period. • A Canadian systematic review explored the effectiveness of reablement and factors that contribute to successful implementation [62]. • Another Canadian systematic review described “the 4R interventions” (reablement, reactivation, rehabilitation, and restorative) with older adults receiving home care to improve “functional abilities, strength, gait speed, social support, loneliness, and the execution of activities of daily living (ADL) and instrumental ADL (IADL)” [63].
Scandinavia (Denmark/Norway/Sweden)	• Although Denmark and Sweden were the earliest adopters of reablement programmes in this region, it was typically referred to as “everyday rehabilitation” or “home rehabilitation”. The first Scandinavian paper to use the term ‘reablement’ discussed a Danish pilot study on whether a home-based reablement programme influenced the ADL ability of older adults [64]. • A Swedish systematic review stated that “re-ablement services are in a period of strong development, but the terms and definitions used remain unclear, and the scientific evidence is still weak” [65]. • A Swedish study was conducted to illuminate older adults’ perceptions of a multi-professional team’s caring skills as success factors for health support in short-term goal-directed reablement [66], and concluded that health professionals’ caring skills need to be addressed as an evidence base in the area of homecare for older people. • A Norwegian RCT on the effectiveness of reablement in home-dwelling older adults [67] and a study protocol for further investigation of the effects described reablement as “an intensive, multidisciplinary, multicomponent, person-centered, home-based type of rehabilitation, where ordinary activities of daily living are used for rehabilitative purposes” [68]. • A Norwegian study on the validity, interpretability, and feasibility of the Canadian Occupational Performance Measure described reablement as a “time limited, person-centered, and goal directed, delivered by a multidisciplinary team” [69]. The authors followed up with a cost-effectiveness analysis alongside an RCT [70]. • The same authors also investigated potential factors that predict an older person’s “occupational performance and satisfaction with that performance at 10 weeks follow-up” [71], and later conducted a clinical controlled trial in 47 Norwegian municipalities on the health effects of reablement in home-dwelling adults, writing that “**reablement** is an emerging approach in **rehabilitation services**, but evidence for its efficacy is rather weak and inconsistent” ([72]; *emphasis added*). • A Norwegian qualitative study explored how an integrated multidisciplinary team experiences participation in reablement [73], followed by a study on how older adults experienced participation in reablement [74]. • Another Norwegian study examined interdisciplinary collaboration [75], and the same authors also described how relatives in a community setting experienced participation in the reablement process [76]. They then conducted a qualitative study on interdisciplinary reablement teams’ roles and experiences and described reablement as “a service for home-dwelling older people experiencing a decline in health and function” [77]. • Other qualitative research from Norway described reablement as an intervention “to provide necessary assistance to the client’s own efforts to achieve the best possible functioning coping ability and participation in social life” [78], as “an approach that aims to assist older adults, irrespective of diagnosis, to continue with their desired activities – as well as the activities of daily living – and to increase their independence” [79], and as “an interprofessional, home-based rehabilitation service that aims to enable senior residents to cope with everyday life and to prevent functional impairments” [80]. • Another Norwegian study wrote “many welfare states offer reablement, also known as restorative care, as an intervention to promote healthy ageing and support older adults in regaining or maintaining their independence in daily life” [81]. • The development of professional practice was the focus of a Norwegian study that presented a cross-sectional descriptive survey of community-working occupational therapists’ involvement in research and development projects [82], which included occupational therapy services and reablement. The authors wrote that “reablement is synonymous to the term ‘restorative care’, which is more commonly used in the USA, and describes home-based, goal-oriented intervention provided by a coordinated multidisciplinary team to home-dwelling elderly with functional decline” [83]. • Another study from Norway explored the content of physiotherapists’ supervision of ‘home trainers’ in reablement teams [83]; the lead author also published a paper that discussed variations in physiotherapy practices across reablement settings [84]. • In an article that examined the practice of support personnel supervised by physiotherapists (PTs) in Norwegian reablement services, the authors focused on PTs’ work, writing that “the key characteristics of the [reablement] service are the short-term and goal-oriented interventions provided by an interprofessional team” [85].
United Kingdom (England/Scotland/Wales) and Ireland	• In discussing a Patient Reported Experience Measure for use by older people in community services, the authors distinguished between social care re-ablement and healthcare hospital-at-home services [86]. • One British article examined ‘re-ablement’ or ‘restorative homecare’ interventions developed as an alternative to reduce dependency in ADLs in homecare to provide “time-limited, intensive input with the specific and explicit aim of enabling people to become independent in personal care activities wherever possible” [87]. • Another article described reablement as a “new paradigm to increase independence in the home amongst the ageing population” [88]. • A systematic review of the evidence on home-care reablement services “found no studies fulfilling our inclusion criteria that assessed the effectiveness of reablement interventions. We did note the lack of an agreed understanding of the nature of reablement” [89]. • In the UK’s first RCT of occupational therapy in homecare reablement, the authors state that The Care Act 2014 statutory guidance considers reablement to be “an example of prevention and has been identified as one of the ‘top-ten’ prevention services for older adults” [90], outlining that these services “aim to assist the person to maximise their ability to carry out activities independently with the aim of reducing the amount of paid care worker input required in the long term” [90]. • A formal examination of reablement stated that “there is limited evidence regarding the organisation and delivery of reablement services in England” [91]. • One study examined family-inclusive approaches to reablement in mental health, and defined reablement in terms of empowerment and social participation, particularly with regards to maximising users’ independence, choice, and quality of life [92]. • Evaluating three reablement services, researchers found a need for greater investment in research on user engagement [93]. • Another article studied goal-orientated cognitive rehabilitation in early-stage Alzheimer’s disease [48]; in a subsequent article, the authors stated that “rehabilitation (or reablement) is grounded in a philosophy of enablement reflecting a positive approach to finding solutions and encouraging optimal functioning. This philosophy emphasises a collaborative approach in service delivery, (…and) translates into specific individualised interventions aimed at optimising functioning [49]. • In a cost analysis of home care reablement for older people, reablement “actively engages the person in activities of daily living, thus improving their ability to perform those activities, which they might have lost after an episode of illness or other adverse life event” [94].
Cross-national studies	• A British–Irish systematic review assessed the effects of time-limited home-care reablement services for maintaining and improving the functional independence of older adults, and stated that “the reablement approach emphasises the active participation of an older person in working towards agreed goals that are designed to maximise independence and confidence” [95]. • British, Danish, Norwegian, and Dutch researchers comprehensively reviewed the reablement approach, describing it as “an intensive, time-limited intervention provided in people’s homes or in community settings, often multi- disciplinary in nature, focusing on supporting people to regain skills around daily activities. It is goal-orientated, holistic and person-centred irrespective of diagnosis, age and individual capacities” [96].

### Quantitative analysis

**Fig. 2:**
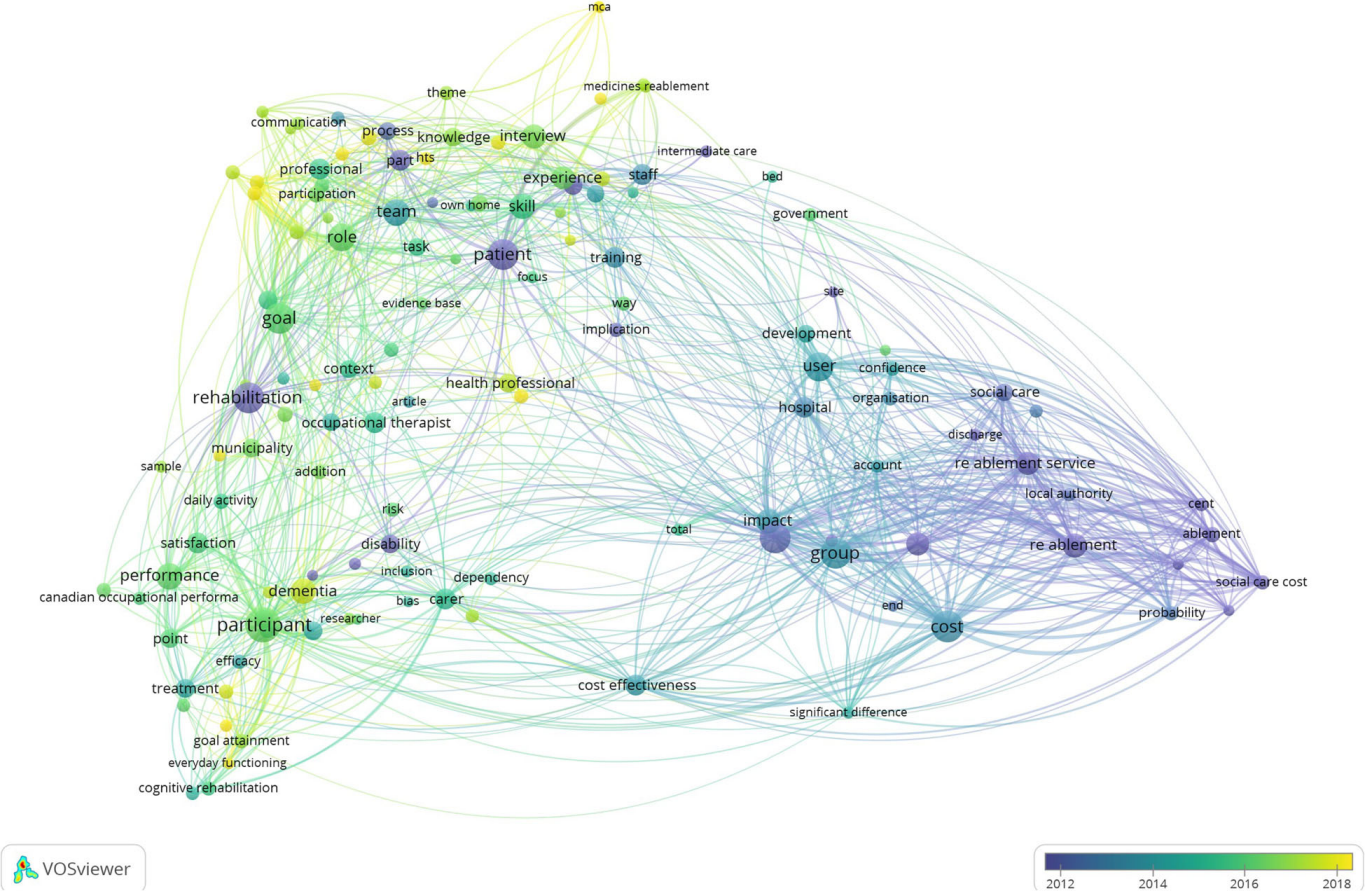
Term Map Visualisation from Abstracts of All 86 Final Eligible Abstracts. We created a term map showing the most relevant terms from the abstracts of all 86 final eligible articles. Out of 2238 terms, 220 met the threshold of five occurrences. Here, we visualised 60% of the most relevant terms, which amounted to 132 terms with 3315 links between the terms. Each circle represents a term from the various abstracts, and the lines connecting the circles represent the interrelatedness of different terms. The size of the circle represents the number of occurrences of the term. The closer the circles are to each other indicates a high co-occurrence of terms representing a topic. The term map is coloured according to publication year, with dark blue/purple circles indicating terms from the earliest publication in 1947 until 2012 transitioning to teal in year 2013–2014, then turquoise/green in 2015–2017, and thereafter yellow indicating terms from the most recent publications in year 2018–2019

We created a term map based on text mining the abstracts of the final eligible articles; see Fig. 2. Our term map shows the most relevant terms from the abstracts of all 86 final eligible articles. Out of 2238 terms, 220 met the threshold of five occurrences. Here, we visualised 60% of the most relevant terms, which amounted to 132 terms with 3315 links between the terms. Each circle represents a term from the various abstracts, and the lines connecting the circles represent the interrelatedness of different terms. The size of the circle represents the number of occurrences of the term. The closer the circles are to each other indicates a high co-occurrence of terms representing a topic. The term map is coloured according to publication year, with dark blue/purple circles indicating terms from the earliest publication in 1947 until 2012 transitioning to teal in year 2013–2014, then turquoise/green in 2015–2017, and thereafter yellow indicating terms from the most recent publications in year 2018–2019.

Based on the number of occurrences of terms emerging in Fig. 2, we can see the central concepts that have been emphasised in the literature throughout the years as listed in Table 2. In the earlier papers from 1947 until 2012, the core principles of reablement had already been conceptualised as a form of rehabilitation for hospitalised patients with disabilities and/or a need for home-care and social care services. In 2013–2014, there appears to be a shift of the target group from ‘patients’ to ‘users’. There was also an emphasis on developing specialised staff training that could create an impact by improving cost-effectiveness, and these programmes were offered as an alternative treatment to usual care. In 2015–2017, there appears to be another shift from ‘users’ to ‘participants’, with special attention to their goals and satisfaction with the programmes. This period also underlined the importance of assessing the performance of reablement services, particularly regarding the health professionals’ and carers’ roles, skills, knowledge, and experience (specifically, nurses and occupational therapists). In the most recent publications from 2018 to 2019, the focus shifted towards how to organise reablement teams and the programme’s overall approach, particularly on improving user involvement and collaboration between healthcare professionals, home-care personnel, and family members. The literature also highlighted an increased interest in making reablement programmes more inclusive to accommodate participants with dementia.

**Table 2:** Number of Occurrences of Terms Emerging from Fig. 2 over Publication Year

1947–2012 Dark Blue - Purple		2013–2014 Teal		2015–2017 Turquoise - Green		2018–2019 Yellow	
Term	N	Term	N	Term	N	Term	N
Home care	38	cost	42	participant	50	dementia	25
Patient	38	group	39	goal	37	health professional	14
Rehabilitation	37	user	32	role	30	reablement team	10
Re ablement	23	team	27	performance	28	home care personnel	9
Re ablement service	22	impact	24	skill	25	kin	9
Social care service	22	staff	18	interview	24	theory	9
Hospital	17	training	18	experience	21	health care professional	8
Part	17	cost effectiveness	17	professional	18	primary outcome	8
Disability	15	treatment	15	carer	18	reablement process	8
Support worker	14	usual care	15	occupational therapist	17	Hts	7
Process	13	development	12	satisfaction	17	reablement approach	7
Social care	13	function	12	knowledge	15	user involvement	7
Ablement	10	supervision	12	nurse	15	family member	6
Implication	9	confidence	9	point	15	resident	6
Probability	9	efficacy	9	control group	14	secondary outcome	6
Social care cost	9	organisation	9	municipality	14	stroke	6
Cent	7	account	7	context	12	everyday functioning	5
Help	7	article	6	task	12	mca	5
Interaction	7	bed	6	caregiver	11	mcas	5
Local authority	7	inclusion	6	participation	11	medication	5
Discharge	6	question	6	daily activity	10		
Home care episode	6	significant difference	6	intervention group	10		
Homecare re ablement service	6	total	6	Norway	10		
Intermediate care	6	semi	5	addition	9		
Ablement service	5			cognitive rehabilitation	9		
Conventional home care service	5			physiotherapist	9		
End	5			risk	9		
Functional status	5			COPM	8		
Relative	5			everyday activity	8		
Site	5			goal attainment	8		
				information	8		
				interdisciplinary collaboration	8		
				theme	8		
				way	8		
				Canadian occupational performance measure	7		
				communication	7		
				early stage dementia	7		
				government	7		
				self	7		
				societal cost	7		
				conversation	6		
				evidence base	6		
				focus	6		
				functional ability	6		
				medicines reablement	6		
				pharmacy technician	6		
				researcher	6		
				sample	6		
				bias	5		
				consumer	5		
				everyday life	5		
				focus group discussion	5		
				Intention	5		
				main theme	5		
				Medline	5		
				own home	5		
				stakeholder	5		

## Discussion

We conducted an exploratory qualitative literature review and quantitative analysis to examine how the concept of ‘reablement’ has been defined and operationalised through time and space. Our scoping review shows that, in the earliest articles dating back to 1947, reablement was understood as a very specific and specialised form of rehabilitation that emphasised ‘resettling’ or ‘restoring’ a person in their local community; i.e., a social model rather than a medical model of recovery. In the earliest versions of reablement, the aim was to help disabled patients restore their functional activities and ‘resettle’ at work and home. As more formalised reablement programmes developed, particularly after 1994, they began to focus on improving independence in basic activities of daily living (ADLs) and the environmental ‘restoration’ of specifically older people. This seems to have led to the development of the ‘restorative-care model’ in the United States during the early 2000s, which incorporated principles adapted from geriatric medicine, nursing, rehabilitation, and goal attainment. At the same time, a ‘reablement’ intervention was being operationalised in the United Kingdom. Both the US and UK approaches were conceptually similar with a focus on supporting older people’s specific goals and needs; moreover, with an emphasis on involving older people themselves in the process of goal-setting, both programmes were meant to be different from traditional (conventional) forms of physical rehabilitation.

By 2011, many Western countries started to implement reablement as an alternative to traditional home-care services for older people. These programmes highlighted the burgeoning ‘reablement philosophy’: “restoring independent functioning rather than resolving health care issues, and helping people to do things for themselves rather than the traditional home care approach of doing things for people that they cannot do for themselves” [33]. Several countries continued to use terms such as ‘reablement’ and ‘restorative home care services’ interchangeably, but the intention of these programmes was generally the same: to assist older people in improving their ability to function in and around the home, and to reduce their need for ongoing home-care services.

As more programmes were implemented worldwide, reablement was typically described as a “time-intensive, time-limited intervention provided in people’s homes or in community settings, often multi-disciplinary in nature, focusing on supporting people to regain skills around daily activities” [62, 95, 96]. In contrast to the pioneering restorative-care model from 2002, such a definition is less focused on goal attainment or social engagement, and more similar to standard definitions of physical rehabilitation. Furthermore, while the overall target population has increasingly become older people, it is most often those “with diverse mortality and morbidity risks, multimorbidity, prognostic outcomes, symptoms, and disability” [89] – a set of circumstances that has long presented significant challenges to healthcare professionals working with more traditional forms of rehabilitation.

Much of the literature in our review states that, since formalised, community-based reablement services for older people first began to be widely implemented in the early 2000s, these programmes lack clear evidence of efficacy, there are inconsistencies in how the programmes are designed and offered in practice, and there is fundamentally a lack of research-based knowledge. This lack of shared understanding and consensus about what reablement *is* or *should be* is reflected in the multitude of terms used to describe nearly identical programmes in different geographical regions. Even in English-speaking countries with similar service models, programmes with similar components have different names: e.g., *reablement* or *re-ablement* (United Kingdom) and the *active service model* or *restorative home support* (Australia, New Zealand, and USA). In Scandinavia, the Swedish version is called *hemrehabilitering* (home rehabilitation), while the term *hverdagsrehabilitering* (everyday rehabilitation) is used in both Norway and Denmark, and the terms *reactivation*, *geriatric rehabilitation*, or *restorative intervention* are sometimes (inconsistently) used in other countries.

Moreover, some countries have recently begun to merge the terms *reablement*, *reactivation*, *rehabilitation*, and *restorative intervention* into the overarching concept of “4R interventions” to define healthcare services for older adults who need support to continue to live at home [63, 84]. By bundling these programmes together under an umbrella term, the implication is that each of these interventions has common features and goals that are relevant to a specific target group; however, the lack of an agreed set of specific “clinical and demographic characteristics makes the target population highly heterogeneous and difficult to define” [84, 89].

Our scoping review of the literature across time and space suggests that, despite definitions, aims, and theoretical aspirations to offer person-centred and goal-directed reablement, very few countries have been able to implement programmes that adequately support an older person’s social involvement or participation in their local community in a safe, culturally sensitive and adaptable way. Frequently, this is due to a lack of infrastructure, professional guidelines, training, and/or resources. Many articles also suggested that, despite already being implemented by national governments, there is a lack of capacity, training, and coordination/cohesion between professional groups to properly implement reablement programmes; in particular, professional training, coordination, and compliance remain problematic. However, even when programmes are implemented, the emphasis is often on improving older people’s physical ability/function and activities of daily living (ADLs) within the home to reduce the costs related to providing homecare and other eldercare services. With a lack of emphasis on individualised goal-setting and supporting social engagement, the reablement philosophy that aims to help older people ‘do things for themselves’ rather than ‘having things done for them’ has simply become a home-based form of physical rehabilitation. As such, the foundational intention to involve older people in setting their own self-identified outcomes and goals continues to be more of a theory than a practice.

### Limitations

Due to the exploratory nature of our study and our main interest in reablement, the search term for all databases was “reablement OR re-ablement”, which resulted in papers published in English by researchers primarily working in Western countries. While this is clearly a limitation, it would also be difficult to interpret the term-map visualisations if there had been a mix of several languages; this could have resulted in multiple words in different languages with the same meaning. Moreover, this means that many countries are not represented in our search because researchers may have published articles in their native language, or they may use different terminology and/or may not describe ‘reablement’ as the concept is generally understood; i.e., the most common (and specific) features are that participants are age 65+, programmes are of a short duration (typically 6–12 sessions) and are provided by paid health professionals, such as physical/occupational therapists or homecare workers, in the participant’s home. A more precise study of how the etymology of different words relates to their specific geographical and/or cultural contexts would be extremely interesting; however, it is beyond the scope of this review. Rather, we have attempted to describe a particular genealogical development and historical trajectory that can be seen in the literature identified in our search.

A limitation of the qualitative analysis was its interpretive approach to reviewing the literature. While we attempted to analyse the texts systematically and objectively with a focus on how ‘reablement’ has been/is being described in specific time periods and geographic regions, it is possible that our analysis reflects certain personal and professional biases. However, we believe that this risk of bias in the qualitative review was mitigated by its overall coherence with the quantitative analysis and term visualisations. Furthermore, our intent with this scoping review was to trace the development of ‘reablement’ as a concept; thus, a more in-depth analysis and discussion about cultural needs for collective enablement or other societal constructions more broadly understood is certainly warranted but beyond the parameters of this review.

## Conclusions

With an emphasis on the older individual’s continuing ability to participate in, contribute to and be productive and valued in society, reablement programmes for older people could be an opportunity for governments to promote a more inclusive and balanced discourse about ‘healthy ageing’. However, with a practice that focuses on *restoration* and *recovery*, old age is often treated as a disability. Our review suggests that, rather than attempting to carefully and methodically implement the first contemporary restorative-care model from 2002 (which was based on principles adapted from geriatric medicine, nursing, rehabilitation, and goal attainment), reablement programmes have been rapidly implemented since the mid-2000s to reduce costs in the eldercare sector without clear evidence for the full scope of their potential effects and outcomes; our review indicates that the long-term efficacy of reablement initiatives still remains weak and inconsistent. Furthermore, in most Western countries, reablement programmes for older people have become interchangeable with standard forms of physical rehabilitation.

As a concept, reablement should be a person-centred approach to home care that recognises the heterogeneity of older people and supports them in their self-defined goals to be *both* more physically independent at home and socially involved in their communities. But until legislators, health professionals, and older people can collectively reach consensus about how person-centred and goal-focused reablement can be more effectively implemented and supported in professional practice, it will be difficult to determine a conceptual description of reablement as a service that is unique, separate, and distinct from more conventional forms of physical rehabilitation. If reablement continues to lack personalisation and fail to recognise older people’s self-defined goals, then these programmes will continue to be less effective and beneficial than they could be. Thus, our review leads us to question the sustainability of reablement programmes in their current iteration – particularly with regards to the ongoing and increasing public-health challenge posed by ageing populations in many Western countries.

## Data Availability

The datasets used and/or analysed during the current study are available from the corresponding author on reasonable request.

## Abbreviations

ADLs: Activities of daily living
COPM: Canadian Occupational Performance Measure
CSED: Care Services Efficiency Delivery
IADL: Instrumental ADL
IFA: International Federation on Ageing
JAMA: *Journal of the American Medical Association*
NHS: National Health Service
PTs: Physiotherapists
PAC: Post-acute care
RCT: Randomised controlled trial
4R interventions: Reablement, reactivation, rehabilitation, and restorative interventions
